# Complete genome sequence of *Streptococcus thermophilus* ST250, a probiotic strain from the NORDBIOTIC collection

**DOI:** 10.1128/mra.00458-26

**Published:** 2026-06-11

**Authors:** Agnieszka K. Szczepankowska, Bożena Cukrowska, Tamara Aleksandrzak-Piekarczyk

**Affiliations:** 1 Institute of Biochemistry and Biophysics, Polish Academy of Sciences (IBB PAS), Warsaw, Poland; 2 Department of Research and Development, Nordic Biotic Ltd., Warsaw, Poland; University of Maryland Baltimore, Baltimore, Maryland, USA

**Keywords:** *Streptococcus thermophilus*, probiotic strain, complete genome sequence, circular chromosome, hybrid genome assembly, Oxford Nanopore sequencing, Illumina sequencing, food isolate

## Abstract

We report the complete genome sequence of *Streptococcus thermophilus* ST250 from the NORDBIOTIC collection, consisting of a single circular chromosome of 1,829,878 bp.

## ANNOUNCEMENT

*Streptococcus thermophilus* includes strains with probiotic properties (1). Strain ST250 degrades immunoreactive gliadin peptides, a trait not previously reported for other *S. thermophilus* strains (2). It was obtained from the NORDBIOTIC collection (Nordic Biotic Ltd., Warsaw, Poland), described as a cheese isolate, and is also deposited in the DSMZ collection (DSM33808). The lyophilized culture was reconstituted in M17 broth (Oxoid) supplemented with 0.5% (wt/vol) glucose (GM17), plated on GM17 agar, and incubated overnight at 37°C under anaerobic conditions.

For DNA extraction, a single colony was inoculated into GM17 broth and grown overnight under the same conditions. Cells were harvested by centrifugation and pretreated with lysozyme (20 mg/mL; Sigma-Aldrich) and mutanolysin (5 U/mL; A&A Biotechnology) for 30 min at 37°C. Genomic DNA was then isolated using a genomic DNA extraction procedure described previously (3), with sodium dodecyl sulfate lysis, proteinase K digestion, and organic extraction. DNA concentration and quality were assessed using a Qubit Fluorometer (Thermo Fisher Scientific), and the sample was subjected to high-quality sequencing using a combined Illumina and Nanopore approach, which enabled accurate base-level correction and resolution of the complete circular chromosome.

Illumina libraries were prepared with the NEBNext Ultra II FS DNA Library Prep Kit (New England Biolabs) and sequenced on a MiSeq instrument (2 × 300 bp, v3 chemistry). Read quality was assessed with FastQC v0.11 and SeqKit v2.8.1, and reads were trimmed and quality filtered with fastp v0.23.4 (4).

For Oxford Nanopore Technologies (ONT) sequencing, high-molecular-weight DNA was sheared using a 26G needle and size-selected to remove fragments of <10 kb using the Short Read Eliminator Kit (Circulomics). A ligation-based library prepared with the Native Barcoding Kit V14 (ONT) was sequenced on a PromethION P2 Solo instrument using an R10.4.1 flow cell. Reads were basecalled with Dorado v7.6.8 (https://github.com/nanoporetech/dorado), trimmed with Porechop_ABI v0.5, filtered with Chopper v0.12.0b, and quality-checked using NanoPlot v1.42 (5, 6).

Long reads were assembled and checked for circularity with Autocycler v0.5.2 (7) using Flye v2.9, Canu v2.3, Raven v1.8.1, Miniasm v0.3-r179, metaMDBG v1.2, NECAT v0.0.1, and NextDenovo v2.5.2 (8–14). The resulting assembly was polished with Illumina reads using PyPOLCA v0.4 and Polypolish v0.6 (15, 16). Default parameters were used for all software. The final sequence was rotated relative to *dnaA* using dnaapler v1.3.0 (17). Final assembly quality was evaluated using QUAST v5.0.2 and Bandage v0.9 (18, 19). The final mean mapped coverage of the chromosome was 294×. Illumina and ONT sequencing statistics are in Table 1.

**TABLE 1:** Statistics of post-processed Illumina and Oxford Nanopore data sets for genome assembly polishing and coverage estimation of *S. thermophilus* ST250^*a*^

Platform	Illumina	ONT
Data type	Read pairs	Reads
Number	4,131,861	70,254
Post-processed bases (bp)	1,229,588,551	320,445,406
N50		6,283

^*a*^ Illumina reads were adapter-trimmed and quality-filtered before polishing and coverage estimation. Mean mapped coverage was calculated after alignment to the final genome assembly.

The final assembly consisted of a circular chromosome of 1,829,878 bp with GC content 39.0%. Annotation with the National Center for Biotechnology Information Prokaryotic Genome Annotation Pipeline v6.10 (20) identified 1,889 genes, including 1,574 protein-coding genes, 226 pseudogenes, 67 transfer RNAs, 18 ribosomal RNAs, 4 non-coding RNAs, and 3 CRISPR arrays.

An *in silico* biosafety screen did not detect antimicrobial resistance determinants, plasmid replicons, or virulence factors listed in the Virulence Factor Database. No canonical genes associated with the production of food-relevant biogenic amines were detected.

## Data Availability

The complete chromosome sequence has been deposited in GenBank under accession no. JBWDMW010000001.1. The associated project and sample are available under BioProject accession no. PRJNA1437409 and BioSample accession no. SAMN56509525, respectively. Raw sequencing reads have been deposited in the Sequence Read Archive under accession nos. SRR37693796 (Illumina) and SRR37693800 (Oxford Nanopore Technologies).
